# Emerging arboviruses in DRC: climate and mobility impacts on dengue, chikungunya, and Zika

**DOI:** 10.1097/MS9.0000000000003812

**Published:** 2025-09-01

**Authors:** Dujardin Makeda, Ekouo Joshua, Christian Tague, Hermann Yokolo, Aymar Akilimali

**Affiliations:** Department of Research, Medical Research Circle (MedReC), Gisenyi, Goma, DR Congo

Arboviruses are viral diseases transmitted by blood-sucking arthropods, mainly mosquitoes of the genus *Aedes* (*A. aegypti* and *A. albopictus*)^[1]^. They constitute a major global public health challenge due to their rapid spread, the co-circulation of several viruses, and limited surveillance and control capacities^[1]^. Among them, dengue, chikungunya, and the Zika virus are experiencing a worrying resurgence, particularly in tropical and subtropical regions such as the Democratic Republic of Congo (DRC), where unplanned urbanization, climate change, and increased trade are favoring their emergence. In the DRC, the hot and humid equatorial climate, combined with high human mobility linked to armed conflicts, cross-border trade, and fragile health infrastructure, creates an environment favorable to the proliferation of vector mosquitoes and the transmission of arboviruses^[1,2]^.

This article aims to analyze the epidemiology of dengue, chikungunya, and Zika in the DRC, in relation to climate change and human mobility. In order to highlight the weaknesses of the health system and propose ways to improve the prevention, surveillance, and management of emerging arboviruses.

Since the late 1990s, several chikungunya epidemics have been reported, notably in Kinshasa and Matadi. In 2011, the outbreak in Matadi was confirmed by laboratory analyses, supported by serological data showing previous circulation of the virus^[2]^. In 2023, the DRC recorded more than 51 million suspected cases of chikungunya, including 28 402 deaths, spread across the provinces of Kinshasa, North Kivu, South Kivu, Ituri, Kasai, Kasai Central, Kwilu, Haut-Katanga, Lomami, Kongo Central, and South Ubangi^[2]^. A serological study conducted between 2021 and 2022 in the communes of Ndjili, Ngaliema, Mont Ngafula, and Limete in Kinshasa revealed a prevalence of 15.3% for dengue, suggesting active circulation of the virus. In contrast, data on the Zika virus remain limited due to a lack of adequate surveillance and diagnosis^[1,2]^.

Climate change plays a key role in the spread of arboviruses^[1]^. In the DRC, rising temperatures, long periods of drought, and irregular rainfall promote the multiplication of *Aedes mosquitoes* by accelerating their life cycle and the extrinsic incubation period of the virus. Flooding caused by intense rains creates numerous breeding sites, allowing epidemics to resume quickly after the wet seasons. These conditions, particularly in densely populated areas such as Kinshasa, Kasai Central, Kwilu, Haut-Katanga, and Kongo Central, prolong the transmission season^[1,3]^.

Furthermore, human mobility contributes significantly to the spread of arboviruses^[3]^. The DRC is facing significant internal displacement due to armed conflict, insecurity, and economic crises. These movements lead to urban overcrowding and the creation of displaced persons camps, promoting close contact and the proliferation of mosquitoes^[1,3]^. Cross-border trade with nine neighboring countries also increases the risks of regional spread, such as between Goma (DRC) and Gisenyi (Rwanda), or between Uvira (DRC) and Bujumbura (Burundi)^[2,3]^.

Despite the magnitude of the risk, the DRC faces serious gaps in health surveillance and response. The country lacks a functioning and extensive entomological network, making early detection of epidemics or the emergence of new vectors difficult. Coordination between laboratories, health authorities, and field actors is insufficient, hampering the reporting and analysis of epidemiological data^[4]^. The lack of rapid diagnostic tools specific to arboviruses limits effective patient care, while public awareness remains low, leading to a lack of understanding of transmission modes and prevention measures^[4,5]^.

Faced with this worrying situation, it is imperative to adopt multisectoral strategies. This involves strengthening integrated surveillance in a *One Health approach*, investing in molecular diagnostic capacities, and decentralizing laboratories^[3,5]^. It is also crucial to train health personnel, involve communities in vector control, particularly through the elimination of larval breeding sites, and integrate climate risk into public health policies^[5]^.

The arboviral situation in the DRC, characterized by active circulation of chikungunya and the increasing emergence of dengue and Zika, is exacerbated by climate change, human mobility, and limited health capacity. The proliferation of *Aedes aegypti* and *A. albopictus* underscores the urgent need for a coordinated response based on research, sustainable financing, regional cooperation, and community engagement.

## Data Availability

Not applicable.
